# Supplementary material for: 4-Coumaroyl-CoA ligases in the biosynthesis of the anti-diabetic metabolite montbretin A
Source: PLoS One. 2021 Oct 7;16(10):e0257478. doi: 10.1371/journal.pone.0257478 (PMC8496819; doi:10.1371/journal.pone.0257478)
Supplement: S1 File — (DOCX) [file pone.0257478.s001.docx]

**Additional file 1.** Protein accession numbers. AAEs that were used in the phylogenetic analysis are listed. AAEs that were also used in the BLASTP search of the montbretia corm time-course transcriptome are bold.

| **Name** | **Clade** | ***Organism*** | **Accession** |
| --- | --- | --- | --- |
| AtLACS1 | I | *Arabidopsis thaliana* | O22898.1 |
| **At4CL1** | **IV** | ***Arabidopsis thaliana*** | [**NP_175579.1**](https://www.ncbi.nlm.nih.gov/protein/NP_175579.1?report=genbank&log$=prottop&blast_rank=2&RID=66UCDYBF013) |
| **At4CL3** | **IV** | ***Arabidopsis thaliana*** | [**NP_176686.1**](https://www.ncbi.nlm.nih.gov/protein/NP_176686.1?report=genbank&log$=prottop&blast_rank=1&RID=66UE9SY6016) |
| Gm4CL1 | IV | *Glycine max* | [AAL98709.1](https://www.ncbi.nlm.nih.gov/protein/AAL98709.1?report=genbank&log$=prottop&blast_rank=1&RID=66U0SUCX016) |
| Gm4CL2 | IV | *Glycine max* | [NP_001236418.1](https://www.ncbi.nlm.nih.gov/protein/NP_001236418.1?report=genbank&log$=prottop&blast_rank=1&RID=66U43HVF016) |
| Gm4CL3 | IV | *Glycine max* | NP_001237270.1 |
| Os4CL1 | IV | *Oryza sativa* | [XP_015650724.1](https://www.ncbi.nlm.nih.gov/protein/XP_015650724.1?report=genbank&log$=prottop&blast_rank=1&RID=66UY9WFK013) |
| Os4CL2 | IV | *Oryza sativa* | [XP_015624111.1](https://www.ncbi.nlm.nih.gov/protein/XP_015624111.1?report=genbank&log$=prottop&blast_rank=1&RID=66V0DHB0013) |
| Pv4CL1 | IV | *Panicum virgatum* | \|  \| [ACD02135.1](https://www.ncbi.nlm.nih.gov/protein/ACD02135.1?report=genbank&log$=prottop&blast_rank=1&RID=66V4SE77013) \| \| --- \| --- \| |
| Pv4CL2 | IV | *Panicum virgatum* | [ADZ96250.1](https://www.ncbi.nlm.nih.gov/protein/ADZ96250.1?report=genbank&log$=prottop&blast_rank=1&RID=66V70C15013) |
| AtOPCL1 | V | *Arabidopsis thaliana* | Q84P21.2 |
| AtACS5 | V | *Arabidopsis thaliana* | Q9LQ12.1 |
| Pn4CL2 | V | *Piper nigrum* | MH078049 |
| SbCLL7 | V | [*Scutellaria baicalensis*](https://www.ncbi.nlm.nih.gov/Taxonomy/Browser/wwwtax.cgi?id=65409) | [AMW91733.1](https://www.ncbi.nlm.nih.gov/protein/AMW91733.1?report=genbank&log$=prottop&blast_rank=1&RID=66V2STYR016) |
| **AtACOS5** | **V** | ***Arabidopsis thaliana*** | **OAP13468.1** |
| **At4g19010** | **V** | ***Arabidopsis thaliana*** | **OAP13468.1** |
| **AtBZO1** | **VI** | ***Arabidopsis thaliana*** | **Q9SS01.1** |
| **AtAAE7** | **VI** | ***Arabidopsis thaliana*** | **Q8VZF1.1** |
| HcCNL | VI | \|  \| [*Hypericum calycinum*](https://www.ncbi.nlm.nih.gov/Taxonomy/Browser/wwwtax.cgi?id=55963) \| \| --- \| --- \| | [AFS60176.1](https://www.ncbi.nlm.nih.gov/protein/AFS60176.1?report=genbank&log$=prottop&blast_rank=1&RID=66UHBPRY016) |
| AtAAE13 | VII | *Arabidopsis thaliana* | [NP_001319565.1](https://www.ncbi.nlm.nih.gov/protein/NP_001319565.1?report=genbank&log$=prottop&blast_rank=1&RID=66VPBD9601R) |
| **AtAAE14** | **VII** | ***Arabidopsis thaliana*** | **AEE31239.1** |
| **AtAAE3** | **VII** | ***Arabidopsis thaliana*** | **Q9SMT7.1** |
