# Supplementary material for: 4-Coumaroyl-CoA ligases in the biosynthesis of the anti-diabetic metabolite montbretin A
Source: PLoS One. 2021 Oct 7;16(10):e0257478. doi: 10.1371/journal.pone.0257478 (PMC8496819; doi:10.1371/journal.pone.0257478)
Supplement: S3 File — (DOCX) [file pone.0257478.s003.docx]

**Additional file 3.** Correlation of *CcAAEs* expression with previously identified MbA pathway genes during young corm development. Pearson correlation coefficients are given and selected candidate genes are bold.

| Name | max correlation | CcUGT1 | CcUGT2 | CcUGT3 | CcUGT4 | CcUGT5 | CcAT1 | CcF3H-2 | CcF3H-1 | CcFLS1 | CcCYP2 | CcCYP1 |
| --- | --- | --- | --- | --- | --- | --- | --- | --- | --- | --- | --- | --- |
| **CcAAE2 (Cc4CL1)** | 0.98 | 0.98 | 0.95 | 0.95 | 0.95 | 0.98 | 0.88 | 0.90 | 0.28 | 0.95 | 0.93 | 0.96 |
| **CcAAE10 (Cc4CL2)** | 0.72 | 0.56 | 0.62 | 0.70 | 0.52 | 0.55 | 0.36 | 0.32 | 0.22 | 0.72 | 0.36 | 0.56 |
| **CcAAE1** | 0.93 | 0.93 | 0.90 | 0.85 | 0.92 | 0.90 | 0.76 | 0.87 | 0.31 | 0.85 | 0.91 | 0.87 |
| **CcAAE3** | 0.91 | 0.78 | 0.69 | 0.62 | 0.76 | 0.79 | 0.83 | 0.91 | 0.27 | 0.62 | 0.91 | 0.79 |
| **CcAAE4** | 0.99 | 0.86 | 0.79 | 0.81 | 0.81 | 0.90 | 0.99 | 0.91 | 0.24 | 0.82 | 0.88 | 0.90 |
| **CcAAE5** | 0.94 | 0.89 | 0.94 | 0.94 | 0.89 | 0.87 | 0.66 | 0.66 | 0.17 | 0.94 | 0.71 | 0.77 |
| **CcAAE7** | 0.97 | 0.96 | 0.92 | 0.90 | 0.95 | 0.97 | 0.95 | 0.93 | 0.15 | 0.89 | 0.92 | 0.90 |
| **CcAAE9** | 0.99 | 0.91 | 0.84 | 0.86 | 0.84 | 0.94 | 0.99 | 0.92 | 0.25 | 0.88 | 0.91 | 0.98 |
| CcAAE6 | 0.55 | -0.02 | -0.03 | 0.09 | -0.11 | 0.01 | 0.14 | 0.05 | 0.55 | 0.09 | 0.02 | 0.08 |
| CcAAE8 | 0.29 | -0.64 | -0.75 | -0.69 | -0.74 | -0.59 | -0.28 | -0.31 | 0.29 | -0.66 | -0.37 | -0.37 |
| CcAAE11 | 0.00 | -0.34 | -0.43 | -0.54 | -0.31 | -0.35 | -0.21 | -0.06 | 0.00 | -0.56 | -0.07 | -0.33 |
| CcAAE12 | 0.10 | 0.09 | 0.09 | -0.04 | 0.19 | 0.08 | 0.06 | 0.10 | -0.55 | -0.07 | 0.10 | -0.05 |
| CcAAE13 | -0.47 | -0.93 | -0.89 | -0.87 | -0.89 | -0.92 | -0.81 | -0.91 | -0.47 | -0.87 | -0.94 | -0.91 |
| CcAAE14 | 0.26 | -0.83 | -0.85 | -0.86 | -0.82 | -0.84 | -0.72 | -0.6 | 0.26 | -0.88 | -0.61 | -0.79 |
| CcAAE15 | -0.33 | -0.98 | -0.97 | -0.93 | -0.97 | -0.96 | -0.83 | -0.91 | -0.33 | -0.93 | -0.93 | -0.89 |
| CcAAE16 | -0.29 | -0.60 | -0.67 | -0.68 | -0.60 | -0.56 | -0.29 | -0.38 | -0.29 | -0.68 | -0.44 | -0.51 |
