# Supplementary material for: 4-Coumaroyl-CoA ligases in the biosynthesis of the anti-diabetic metabolite montbretin A
Source: PLoS One. 2021 Oct 7;16(10):e0257478. doi: 10.1371/journal.pone.0257478 (PMC8496819; doi:10.1371/journal.pone.0257478)
Supplement: S5 File — (DOCX) [file pone.0257478.s005.docx]

**Additional file 5.** Activity and expression of AAEs. (A) Activity of heterologously expressed AAEs with different hydroxycinnamic acid substrates. + enzyme activity detected, - no enzyme activity detected. (B) Protein gel blot showing AAE expression. Ni-purified proteins were used with and detected with monoclonal AntipolyHistidine-Alkaline Phosphatase antibody and the 1-step NBT/BCIP substrate solution

| **A** | **Cinnamic acid** | ***p*-coumaric acid** | **Caffeic acid** | **Ferulic acid** | **Sinapic acid** |
| --- | --- | --- | --- | --- | --- |
| Cc4CL1 | - | **+** | **+** | **+** | - |
| Cc4CL2 | **+** | **+** | **+** | **+** | - |
| CcAAE1 | - | - | - | - | - |
| CcAAE3 | - | - | - | - | - |
| CcAAE4 | - | - | - | - | - |
| CcAAE5 | - | - | - | - | - |
| CcAAE7 | - | - | - | - | - |
| CcAAE9 | - | - | - | - | - |

**B**


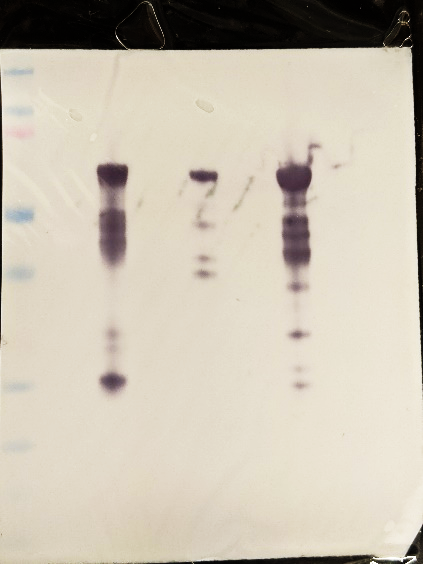

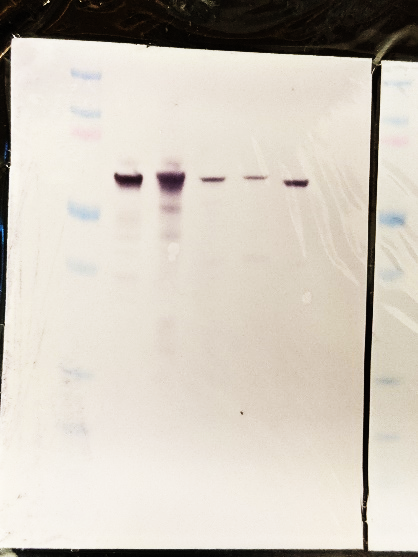


CcAAE2 (Cc4CL1)

CcAAE10 (Cc4CL2)

CcAAE3

CcAAE4

CcAAE5

CcAAE9

CcAAE7

kDa

75

50

37

75

50

37

kDa

CcAAE1
