# Supplementary material for: 4-Coumaroyl-CoA ligases in the biosynthesis of the anti-diabetic metabolite montbretin A
Source: PLoS One. 2021 Oct 7;16(10):e0257478. doi: 10.1371/journal.pone.0257478 (PMC8496819; doi:10.1371/journal.pone.0257478)
Supplement: S6 File — (DOCX) [file pone.0257478.s006.docx]

**Additional file 6.** Combinations of genes used for transient expression in *N. benthamiana*. Each gene was individually expressed as a *35S_pro_*:gene construct and genes were combined by mixing equal volumes of Agrobacterium cultures for infiltration.

| **Combination** | **Constructs** |
| --- | --- |
| MBG | *CcFLS + CcCYP2 + CcMYB4 + CcUGT1 + CcUGT2 + CcAT1 + CcUGT3 + CcUGT4 + CcUGT5* |
| MBG+Cc4CL1 | *CcFLS + CcCYP2 + CcMYB4 + CcUGT1 + CcUGT2 + CcAT1 + CcUGT3 + CcUGT4 + CcUGT5 +* ***Cc4CL1*** |
| MBG+Cc4CL2 | *CcFLS + CcCYP2 + CcMYB4 + CcUGT1 + CcUGT2 + CcAT1 + CcUGT3 + CcUGT4 + CcUGT5 +* ***Cc4CL2*** |
| MBG+CcAAE1 | *CcFLS + CcCYP2 + CcMYB4 + CcUGT1 + CcUGT2 + CcAT1 + CcUGT3 + CcUGT4 + CcUGT5 +* ***CcAAE1*** |
| MBG+CcAAE3 | *CcFLS + CcCYP2 + CcMYB4 + CcUGT1 + CcUGT2 + CcAT1 + CcUGT3 + CcUGT4 + CcUGT5 +* ***CcAAE3*** |
| MBG+CcAAE4 | *CcFLS + CcCYP2 + CcMYB4 + CcUGT1 + CcUGT2 + CcAT1 + CcUGT3 + CcUGT4 + CcUGT5 +* ***CcAAE4*** |
| MBG+CcAAE5 | *CcFLS + CcCYP2 + CcMYB4 + CcUGT1 + CcUGT2 + CcAT1 + CcUGT3 + CcUGT4 + CcUGT5 +* ***CcAAE5*** |
| MBG+CcAAE7 | *CcFLS + CcCYP2 + CcMYB4 + CcUGT1 + CcUGT2 + CcAT1 + CcUGT3 + CcUGT4 + CcUGT5 +* ***CcAAE7*** |
| MBG+CcAAE9 | *CcFLS + CcCYP2 + CcMYB4 + CcUGT1 + CcUGT2 + CcAT1 + CcUGT3 + CcUGT4 + CcUGT5 +* ***CcAAE9*** |
