# Supplementary material for: 4-Coumaroyl-CoA ligases in the biosynthesis of the anti-diabetic metabolite montbretin A
Source: PLoS One. 2021 Oct 7;16(10):e0257478. doi: 10.1371/journal.pone.0257478 (PMC8496819; doi:10.1371/journal.pone.0257478)
Supplement: S7 File — (DOCX) [file pone.0257478.s007.docx]

**Additional file 7.** Montbretin A, B and C levels in *N. benthamiana* plants transiently expressing MBGs and AAE. Mean (n = 4) and SE are shown. See Additional file 6 for combination of genes used for infiltration. An ANOVA was used to test for significant differences. MbA (F_8,18_ = 1.57, p = 0.20), MbB (F_8,18_ = 1.43, p = 0.25), MbC (F_8,18_ = 1.06, p = 0.43)

0

100

200

300

400

MBGs

MBGs + Cc4CL1

MBGs + Cc4CL2

MBGs + CcAAE1

MBGs + CcAAE3

MBGs + CcAAE4

MBGs + CcAAE5

MBGs + CcAA7

MBGs + CcAAE9

µg * g^-1^ (FW)

MbA

MbB

MbC
