# Supplementary material for: 4-Coumaroyl-CoA ligases in the biosynthesis of the anti-diabetic metabolite montbretin A
Source: PLoS One. 2021 Oct 7;16(10):e0257478. doi: 10.1371/journal.pone.0257478 (PMC8496819; doi:10.1371/journal.pone.0257478)
Supplement: S8 File — (DOCX) [file pone.0257478.s008.docx]

**Additional file 8.** Identification of montbretins produced by *N. benthamiana* transiently expressing MBGs. (A) Extracted ion chromatogram (EIC) of MbA (peak 2, m/z 1227.5), MbB (peak 1, m/z 1211.5), and MbC (peak 3, 1241.5) MS/MS fragmentation patterns of MbA (B), MbB (C), MbC (D) and MS^3^ of MbC (m/z 1241.5 🡪 963.0) (E).

787

0

2

4

6

Relative intensity (EIC x 100,000)

200

600

1000

m/z

MS/MS 963.0

**B**

Relative intensity (EIC x 100,000)

787

949

1065.4

0.0

0.2

0.5

0.7

1.0

1.2

400

800

1200

m/z

MS/MS 1227.5

MbA

★

MbB

★

Relative intensity (EIC x 100,000)

787

933

1065

0

2

4

6

200

600

1000

m/z

★

**C**

Relative intensity (EIC x 10,000)

787

963

0

2

4

6

8

200

600

1000

m/z

MS/MS 1241.5

MbC

★

**D**

**E**

MbC-XR

MS/MS 1211.5

Relative intensity (EIC x 100 000)

**A**

3

2

1

EIC 1211.5

EIC 1241.5

EIC 1227.5

Retention Time (min)

9.0

8.0

7.0

6.0

5.0

1

3

1

3

10

20
