# Supplementary material for: 4-Coumaroyl-CoA ligases in the biosynthesis of the anti-diabetic metabolite montbretin A
Source: PLoS One. 2021 Oct 7;16(10):e0257478. doi: 10.1371/journal.pone.0257478 (PMC8496819; doi:10.1371/journal.pone.0257478)
Supplement: S9 File — (DOCX) [file pone.0257478.s009.docx]

**Additional file 9.** Parts of the MoClo system (Addgene, MA, USA) used for the construction of *S. cerevisiae* expression modules. Modules were assembled using the Golden-gate cloning pipeline described by Lee and co-workers (2015).

| Position 1 | spacer, ConLS, ConR1 |
| --- | --- |
| Position 2 | *CcCHS2*, ConL1, ConR2, *pHTB2*, *tENO1* |
| Position 3 | *CcCHI2*, ConL2, ConR3, *pRPL18B*, *tSSA1* |
| Position 4 | *4CL*, ConL3, ConR4, *pHHF1*, *tTDH1* |
| Position 5 | spacer, ConL4, ConRE |
| Vector backbone | ConLS', ConRE', CEN6/ARS4, Kan-ColE1, *URA3* |
