# Supplementary material for: 4-Coumaroyl-CoA ligases in the biosynthesis of the anti-diabetic metabolite montbretin A
Source: PLoS One. 2021 Oct 7;16(10):e0257478. doi: 10.1371/journal.pone.0257478 (PMC8496819; doi:10.1371/journal.pone.0257478)
Supplement: S10 File — (DOCX) [file pone.0257478.s010.docx]

**Additional file 10**. Identification of products formed by *S. cerevisiae* expressing a flavanone biosynthetic module. Peak 1 and peak 3 produced by *S. cerevisiae* as shown in Figure 6 were tentatively identified based on their MS/MS fragmentation patterns compared to fragmentation patterns described by Waki *et al*., 2020 [1]. Peak 1, tentatively identified as *p*-coumaroyltriacetic acid lactone; peak; peak 3, tentatively identified as caffeoyltriacetic acid lactone.

125

145

185

201

227

1

3

75

125

175

225

m/z

★

MS/MS 271

Peak 1

5

★

Peak 3

125

152

161

203

217

243

1

3

75

125

175

225

275

m/z

MS/MS 287

Relative intensity (EIC x 1 000)

1. Waki T, Mameda R, Nakano T, Yamada S, Terashita M, Ito K, Tenma N, Li Y, Fujino N, Uno K *et al*: A conserved strategy of chalcone isomerase-like protein to rectify promiscuous chalcone synthase specificity. *Nature com* 2020, 11(1):870.
