# Supplementary material for: 4-Coumaroyl-CoA ligases in the biosynthesis of the anti-diabetic metabolite montbretin A
Source: PLoS One. 2021 Oct 7;16(10):e0257478. doi: 10.1371/journal.pone.0257478 (PMC8496819; doi:10.1371/journal.pone.0257478)
Supplement: S11 File — (DOCX) [file pone.0257478.s011.docx]

**Additional file 11.** Oligonucleotides used in this study.

| **Gene** | **Forward (5’ - 3’)** | **Reverse (5’ - 3’)** |  |
| --- | --- | --- | --- |
| CcAAE1 | ATGTCACCCTCCATTGATCCCAAC | CTACAATTTGGGATTCAATTTCGAGGA |  |
| CcAAE2 (Cc4CL1) | ATGATCACTATAGCAACTCATGAAGCTC | TTAAGTAGAAACAACCACTCTTGCCC |  |
| CcAAE3 | ATGGCAGTCTTGGAAGACAATGGCT | TCATAGCCTAGCTGAGATCTCTAGC |  |
| CcAAE4 | ATGGCAAACTTGGCCTGCTTTGATA | CTACAGTTGGTCACTCCCTTTTTTATTG |  |
| CcAAE5 | ATGTCTTCTTCTTCCTCCATAGATCC | TCAGTAGTAGAATGGTACTTCTGTGGA |  |
| CcAAE7 | ATGTCGCCCTCCTCCATTGAC | CTACAATTTAGGATTCGTTGAGGATGCT | |
| CcAAE9 | ATGGGAGAGTTCGCAGTACGTAAAG | ATAGGCCTGCCTCTTGGCCAAAT |  |
| CcAAE10 (Cc4CL2) | ATGGGTTCCATTCCTTCGGAGAAAG | TCACAGCTGCTGCCCCTTAG |  |
