# Supplementary material for: 4-Coumaroyl-CoA ligases in the biosynthesis of the anti-diabetic metabolite montbretin A
Source: PLoS One. 2021 Oct 7;16(10):e0257478. doi: 10.1371/journal.pone.0257478 (PMC8496819; doi:10.1371/journal.pone.0257478)
Supplement: Attachment — Submitted filename: 00 Response to Reviewer comments 083021.pdf [file pone.0257478.s012.pdf]

Dear Editor:

We have very much appreciated yours and the reviewers' very positive assessment of our manuscript, which you recommended to be accepted for publication in PLOS ONE on August 26.

We have addressed the specific comments with the requested minor revisions as detailed below.

Thank you very much again for your very fast and positive review process.

Best regards,

Joerg Bohlmann

Reviewer 1

It is possible that the product pattern in Crocosmia is result of a distinct substrate pattern, i.e., much higher concentration of caffeic acid. What are the specific expression pattern of HCT, Hydroxycinnamoyl CoA shikimate:quinate hydroxycinnamoyltransferase, C3'H, p-coumaroyl shikimate 3'-hydroxylase and CSE, caffeoyl shikimate esterase? These are possibly driving the caffeate formation.
Indeed, this is a question that we are currently exploring with new work. We do not yet have the data to speculate on this question.

The Arabidopsis 4CL2 isoform was shown to have higher activity toward caffeate and was also investigated for its substrate specificity, albeit against sinapate.
We added wording in the Discussion about At4CL2 and a reference for Costa et al. (2005) [29].

I strongly suggest to have cytochromes P450 enzymes annotated by David Nelson, instead of using a generic nomenclature.
CcCYP2 was annotated by Dr. Nelson as CYP75B138 (Irmisch et al. 2019b). We have added this annotation at the first mention of this P450.

L 193, symbol font
corrected

L 472, font color
corrected

Phylogeny, I suggest collapsing branches with less than 50% bootstrap support.
There are two instances just below 50%. We believe it is more informative to keep the branches separated with the bootstrap values shown.

Fig. 6, I suggest labelling the tentatively identified products in panel C in the schematic representation B.
Done. The products were indeed labelled in panel B matching the peak numbers in panel C. However, the labels 1 and 3 were not in bold, which is why this may not have been obvious.

Accession numbers are missing.
Accession numbers are now included.

Reviewer 2

The title may be perceived as misleading, because no functional (e.g. reverse genetic) data are presented that prove involvement of the 4CLs in montbretin biosynthesis. It may also be an overstatement that MbA is an anti-diabetic; without clinical studies are published, it should rather be considered a candidate.
We removed the word "involved". The word "anti-diabetic" is justified, as this activity was proven in the pre-clinical animal studies with diabetic rats.

The supporting information show a Western lot highlighting successful heterologous expression of the enzymes tested. It is unclear what antibody has been used (anti-His?) and if protein crude extracts have been used or purified proteins as the

method is not described in the M&M section. It is noteworthy that 4CL1 and 4CL2 had highest expression levels (and the largest amounts of unspecific labelling); is it just a coincidence that the only two active enzymes also expressed best?
We added the requested information was to the caption (additional File 5). The information was also already included in the methods.

NCBI accession numbers for the 4CLs are to be published (replace 'xxx' with actual accession numbers). It would also be good to provide accession numbers for the previously published transcriptome assembly and transcript count data.
Accession numbers are now included.

To determine substrate specificity, it would have been better to determine enzyme kinetic properties, especially given that simple photometric assays are available to test for 4CL activity. This would have given a more accurate comparison compared to the competition assays used. However, I acknowledge that protein stability of purified 4CL enzymes is a frequently observed problem rendering it difficult to determine extensive kinetic properties.
We tried the photometric methods, but were not successful due to protein stability and sensitivity issues.

You discuss that using a caffeate-specific 4CL would be an intuitive way to increase MbA production in Nicotiana and suggest targeted or directed evolution techniques to increase substrate-specificity. I agree but would suggest to start with a 4CL that already has a preference for caffeate; I think this is true for one of the Arabidopsis isoforms.
This is very good suggestion for the future work. We added wording in the Discussion about At4CL2 and a reference for Costa et al. (2005) [29].

It remains unclear to me why it would be important to establish the early steps of flavonoid biosynthesis in yeast using the Montbretia enzymes when similar approaches using genes from other plants were already successful. Wouldn't it be more efficient to build on these published successes and add the montbretin specific part to these strains that already produce basic flavonoids? I assume this relates to the ability to protect the engineered strains once or if efficient MbA production in yeast has been achieved making it commercially viable to produce it this way?
There are indeed good reasons to consider both established and new flavonoid producing yeast strains, which would be outside of the discussion of this paper.

Concluding Response: Again, we very much appreciated the very positive assessment of our manuscript and the suggestions for minor revisions and corrections.
